# HIV-1 Tat Protein Induces Production of Proinflammatory Cytokines by Human Dendritic Cells and Monocytes/Macrophages through Engagement of TLR4-MD2-CD14 Complex and Activation of NF-κB Pathway

**DOI:** 10.1371/journal.pone.0129425

**Published:** 2015-06-19

**Authors:** Nawal Ben Haij, Rémi Planès, Kaoutar Leghmari, Manutea Serrero, Pierre Delobel, Jacques Izopet, Lbachir BenMohamed, Elmostafa Bahraoui

**Affiliations:** 1 INSERM, U1043, Toulouse, France, CNRS, U5282, Toulouse, France; 2 Université Paul Sabatier Toulouse, Toulouse, France; 3 Department of Infectious Diseases, Toulouse University Hospital, Toulouse, France; 4 Laboratory of Cellular and Molecular Immunology, Gavin Herbert Eye Institute, University of California Irvine, School of Medicine, Irvine, CA, 92697, United States of America; 5 Institute for Immunology, Irvine, CA, 92697, United States of America; 6 Department of Molecular Biology & Biochemistry, University of California Irvine, School of Medicine, Irvine, CA, 92697, United States of America; University of Leuven, Rega Institute, BELGIUM

## Abstract

We recently reported that the human immunodeficiency virus type-1 (HIV-1) Tat protein induced the expression of programmed death ligand-1 (PD-L1) on dendritic cells (DCs) through a TLR4 pathway. However, the underlying mechanisms by which HIV-1 Tat protein induces the abnormal hyper-activation of the immune system seen in HIV-1 infected patients remain to be fully elucidated. In the present study, we report that HIV-1 Tat protein induced the production of significant amounts of the pro-inflammatory IL-6 and IL-8 cytokines by DCs and monocytes from both healthy and HIV-1 infected patients. Such production was abrogated in the presence of anti-TLR4 blocking antibodies or soluble recombinant TLR4-MD2 as a decoy receptor, suggesting TLR4 was recruited by Tat protein. Tat-induced murine IL-6 and CXCL1/KC a functional homologue of human IL-8 was abolished in peritoneal macrophages derived from TLR4 KO but not from Wt mice, confirming the involvement of the TLR4 pathway. Furthermore, the recruitment of TLR4-MD2-CD14 complex by Tat protein was demonstrated by the activation of TLR4 downstream pathways including NF-κB and SOCS-1 and by down-modulation of cell surface TLR4 by endocytosis in dynamin and lipid-raft-dependent manners. Collectively, these findings demonstrate, for the first time, that HIV-1 Tat interacts with TLR4-MD2-CD14 complex and activates the NF-κB pathway, leading to overproduction of IL-6 and IL-8 pro-inflammatory cytokines by myeloid cells from both healthy and HIV-1 infected patients. This study reveals a novel mechanism by which HIV-1, via its early expressed Tat protein, hijacks the TLR4 pathway, hence establishing abnormal hyper-activation of the immune system.

## Introduction

Persistent HIV-1 infection is associated with abnormal hyper-activation of the immune system and the expression of multiple immunosuppressive factors including interleukin-10 (IL-10) [1,2], programmed death ligand-1 (PD-L1), programmed death receptor 1 (PD-1) [3–5] and indoleamine 2,3 dioxygenase (IDO) [6]. Each of these immunosuppressive factors contributes to the impairment of the development of efficient protective immunity. HIV-1 persistence is associated with various physiological dysregulation and leads inevitably to a progressive depletion of CD4^+^ cells [7]. Additional abnormalities include neurological disorders such as HIV-1 associated dementia (HAD) [8] and cell proliferative dysfunctions associated with the development of various cancers [9–11]. The majority of these pathological disorders are facilitated by the capacity of HIV-1, through its various viral components, to disturb the physiological cytokine network [12,13]. Accordingly, during the course of HIV-1 infection, an increase in the production of pro-inflammatory cytokines, including TNF-α, IL-1β, IL-6 and IL-8, is associated with the activation of HIV-1 viral replication, and the progression to AIDS [14–17]. Thus, it is essential to determine the viral components responsible for the inflammatory response and to understand the underlying mechanisms and signaling pathways. Several studies have reported the role of HIV-1 proteins, including gp120 [18–21], Tat [22–25], Nef [26–28] and Vpr [29,30] gene products in the immune system dysregulation seen during HIV-1 persistent infection. Some ss-RNA domains expressed by the HIV-1 genome, and HIV-1 gene products act as PAMPs targeting membrane and cytoplasmic PRRs, including TLR2 [31], TLR3 [32], TLR4 [29,33], TLR7 [34], TLR8 [35,36] and RIG-1 [37].

Given the ability of HIV-1 to stimulate the production of significant amounts of IL-6 and IL-8 pro-inflammatory cytokines, in a recent study we undertook experiments: (*i*) to determine whether Tat protein was among the viral components responsible for such inflammatory cytokine over-production; (*ii*) to understand the underlying cellular mechanisms; and (*iii*) to determine the intracellular signaling pathways involved. Tat protein is an early regulatory protein of 14–16 kDa that has a variable length of 86 to 104 amino acids. This protein plays an essential role in the transactivation activity of HIV-1 promoter [38]. Its deletion totally abrogates the replicative capacity of the virus. By recruiting several cellular proteins, including cyclin T1 and CDK9 at the TAR region of nascent viral transcripts Tat leads to the hyper-phosphorylation of RNA-Pol-II, a modification that is crucial for the restoration of its elongation function. Among many potential HIV-1 components, we chose HIV-1 Tat protein as a potential candidate because of its pivotal role in the physiopathology of HIV-1 infection. In addition to its crucial role in the viral cycle, several additional functions have been attributed to HIV-1 Tat protein including: (*i*) stimulation of pro-inflammatory cytokine production [23,24,39–41], (*ii*) establishment of an immunosuppressive state through the induction of PD-L1 [42], IDO [43,44] and IL-10 [22,33,45–47] production associated with a dysfunction in T cell responses, as we recently reported [42,43]; (*iii*) contribution to the spread of HIV-1 and to pathogenesis [10,39,48–51].

The virological function of HIV-1 Tat protein operates mainly inside infected cells, mostly in the nucleus [52]. In contrast, the immunological function of HIV-1 Tat protein appears to be attributable to its extracellular effects on non-infected immune cells, such as DCs and monocytes/macrophages [53,54]. During acute HIV-1 infection, Tat protein has been found at nano-molar levels: (*i*) in the sera of infected patients [55–57]; and (*ii*) in the supernatant of T-cell cultures infected *in vitro* with HIV-1 [58,59]. We hypothesize that the extracellular HIV-1 Tat protein interacts with, and is then taken up by, neighboring DCs and monocytes/macrophages, regardless of whether they are infected or not. Such interaction may lead to induction of pro-inflammatory mediators contributing to the abnormal hyper-activation of the immune system seen in HIV-1 infected patients.

We have previously reported that HIV-1 Tat protein induced TNF-α and IL-10 production by monocytes [22,41,60–63]. This production is dependent on the activation of PKC-βII and PKC-δ isoforms and involves classical and alternative NF-κB pathways [64]. More recently, we have shown that Tat-induction of TNF-α and IL-10 production in human monocytes is inhibited in the presence of blocking anti-TLR4 antibodies [33]. Tat protein is also able to interact in a solid phase assay with soluble recombinant TLR4-MD2 complex [33]. However, the underlying mechanisms by which HIV-1 Tat protein induces this abnormal hyper-activation remain to be fully elucidated. Despite these indirect characterizations, more direct approaches are required to demonstrate the effect of Tat on: (*i*) the modulation of the expression of TLR4 on the cell surface; (*ii*) its capacity to activate the key transcription factor NF-κB pathway; and (*iii*) the induction of other pro-inflammatory cytokines, such as IL-6 and IL-8.

In this study, we show that HIV-1 Tat protein was able to induce production of the pro-inflammatory cytokines IL-6 and the pro-inflammatory/chemoattractant IL-8 by DCs and primary human monocytes in both healthy and HIV-1 infected patients. Using HEK cells stably transfected with TLR4-CD14-MD2 and peritoneal macrophages from TLR4 KO mice, we demonstrated that Tat protein activated the NF-κB pathway in a TLR-4-dependent manner. Moreover we demonstrated that, upon activation of the TLR4 pathway, Tat triggered negative regulatory mechanisms including induction of SOCS-1 protein expression and down-modulation of cell surface TLR4 by endocytosis in dynamin and lipid-raft-dependent manners.

## Materials and Methods

### Ethics statement

This study on human cells was approved by the Research Ethical Comity Haute-Garonne. Human Peripheral blood mononuclear cells were isolated from buffy coat, from human donors. Buffy coats were provided anonymously by the EFS (établissement français du sang, Toulouse, France). Written informed consents were obtained from the donors under EFS contract N° 21/PVNT/TOU/INSERM01/2011-0059, according, to “Decret N° 2007–1220 (articles L1243-4, R1243-61)”. The study, using mice as animal models, was conducted in accordance with the EU regulations and with the French national chart for ethics of animal experiments (articles R214-87 to 90 of the code rural). The protocol was approved by the committee on the ethics animal experiments of the Region Midi-Pyrénées and by IFR 150 (permit numbers: 04-U563-DG-06 and MP/18/26/04/04). To minimize suffering, all animals were handeled under anesthezia.

### Human monocytes isolation

PBMCs were isolated from buffy coat of healthy HIV-1-negative donors (EFS Toulouse Purpan, France) or from the blood samples of HIV-1 infected patients under antiretroviral treatment (from the department of infectious diseases of Toulouse University Hospital) by Ficoll density gradient. Briefly, PBMC were counted and re-suspended in complete medium containing 60% AIMV, 30% Iscove (Gibco) with 10% foetal calf serum (FCS), penicillin (100 IU/mL) and streptomycin (100 μg/ml). Monocytes were separated from lymphocytes by adherence to tissue culture plastic (Beckton Dickinson). After an incubation of 1 h at 37°C 5% CO_2_, non-adherent cells were removed and adherent cells (> 94% CD14^+^ by flow cytometry analysis) were washed and cultured in a complete medium containing 10% FCS, penicillin (100 IU/ml) and streptomycin (100 μg/ml) before the experiments. In some experiment (as indicated in the figure legend) monocytes were isolated from PBMC by positive selection using CD14 MicroBead according to the manufacturer instruction (Miltenyi Biotec). This technique give rise to a purity of 99% CD14^+^ cells by flow cytometry analysis.

### Human embryonic kidney 293-cell line

Transfected HEK cell line stably expressing TLR4, TLR4-CD14-MD2, TLR2-CD14 and carrying an empty plasmid HEK cell line (HEK-Null) were purchased from Invivogen. HEK-Null and HEK-TLR4 cell lines were cultured in DMEM supplemented with 10% FCS, normocin (100 μg/mL) and blasticidin (10 μg/ml) while HEK TLR4-CD14-MD2 and HEK TLR2-CD14 cell lines were grown in DMEM 10% FCS, normocin (100 μg/ml), blasticidin (10 μg/ml) and hygrogold (50 μg/ml) at 37°C and 5% CO_2_.

### Human pro-monocytic cell line

U937 cells were obtained from the American Type Culture Collection (ATCC) and grown in RPMI1640 supplemented with 10% FCS, penicillin (100 IU/ml) and streptomycin (100 μg/ml) and maintained in a 37°C humidified atmosphere with 5% CO_2_.

### Primary mouse peritoneal macrophages

C57BL/6 mice were purchased from Charles Rivers. C57BL/6 mice (Wt.) and TLR2 and TLR4 Knockout (KO) with a C57BL/6 strain background (TLR2^-/-^ and TLR4^-/-^) were a generous gift from Dr. B. Ryffel (laboratory of molecular immunology and embryology, CNRS, Orléans, France). Primary macrophages were isolated as previously described [65]. Briefly, mice were injected intra peritoneally with 1 ml of thioglycolate medium 3% (Biomerieux). Three days later, the mice were sacrificed and macrophages were recovered by peritoneal washes and then enriched by adherence selection for 1 h in complete medium: DMEM supplemented with 2% FCS, penicillin (100 IU/ml) and streptomycin (100 μg/ml). Isolated macrophages were > 95% CD11b+ by flow cytometry analysis.

### Generation of monocyte-derived DCs

To allow their differentiation into monocyte-derived DCs (MoDCs), cells were cultured in RPMI medium (Invitrogen) supplemented with 10% FCS (Invitrogen), 100 IU/ml penicillin, 100 μg/ml streptomycin, 10 ng/ml GM-CSF and 10 ng/ml IL-4 (HumanZyme). After 5 days of culture, loosely adherent cells were recovered by gentle pipetting and used as immature DCs in our experiments. Over 90% of cells had the standard phenotype of immature DCs: CD1a+, CD14-, CD80+, CD86+, CD83-, HLA-DR+.

### Isolation of primary human myeloid DCs

Primary myeloid DCs were isolated from human PBMCs by negative selection, using the Myeloid Dendritic Cell Isolation Kit, according to the manufacturer’s instructions (Miltenyi Biotech). The isolated DCs were characterized by the expression of CD1c (BDCA-1) marker.

### Tat protein and TLR ligands

Tat proteins: Recombinant HIV-1 Tat protein 1–86 was obtained from “Agence Nationale de la Recherche sur le SIDA” (Paris, France). Recombinant GST, GST-Tat 1–101 and Tat deleted mutant (Tat 1–45, Tat 30–72) proteins were produced in our laboratory. Each protein was produced and purified as previously described [22]. The level of endotoxin contamination was assessed using the Limulus amebocyte lysate assay (Bio-Sepra, Villeneuve la Garenne, France). All of these recombinant proteins contained less than 0.3 EU/μg LPS, the limit of detection of this test. Chemically synthesized Tat (aa 1 to 86) protein from the HIV-1 Lai strain were produced as described elsewhere [66]. TLR agonist lipopolysaccharide (LPS) (TLR4) from E. *coli*, serotype R515, was purchased from Alexis biochemicals. Pam_3_CSK_4_ (TLR2/1 ligand) was purchased from Invitrogen. Phorbol 12 myristate-13-acetate (PMA), a PKC activator, was purchased from Calbiochem. Recombinant human TLR4/MD2 soluble proteins were purchased from R&D.

### Antibodies and chemical inhibitors

Inhibition experiments: monoclonal antibodies against human antigens: anti-TLR4 (clone HTA125), anti-TLR2 (clone TL2.1) and mouse IgG2a isotype were purchased from eBioscience. Monoclonal anti-Tat antibodies were obtained from ANRS (Paris, France), clone T-A 9, isotype IgG2a that recognize a linear epitope in the N-terminal region 1–15 (ANRS Catalog, page IX-101, 1992). The raft disrupting drug methyl–β-cyclodextrin (M-βCD) and a GTPase dynamin inhibitor dynasore were purchased from Sigma-Aldrich. The NF-κB inhibitor (Bay11-7082) was purchased from Calbiochem.

Plasmids: The following plasmids psiRNA-hTLR4, psiRNA-hTLR2, pZERO-hTLR2, pzero-hTLR4, pORF-LacZ and pNiFty2-SEAP were purchased from Invivogen.

Antibodies for flow cytometry analysis: goat anti-human TLR4, were purchased from R&D System. Unlabeled mouse anti-TLR4 and labeled mouse anti-TLR4-PE antibodies were purchased from eBioscience. Mouse isotype control IgG2a–PE was purchased from eBioscience. FITC coupled secondary antibodies produced against mouse immunoglobulins were obtained from DAKO. Anti-CD14-APC, anti-CD3-Pacific Blue, anti-IL-8 PE, anti-IL6-FITC and isotype control (IgG1-APC) were purchased from Ozyme.

Antibodies for Western Blot analysis: monoclonal anti-human TLR2 and anti-TLR4 were from R&D Systems. Rabbit polyclonal anti-IkBα, anti-p65 and mouse monoclonal anti-p50, anti-TFIIB were all purchased from Santa Cruz Biotechnology. For loading control, monoclonal anti-β-actin (clone AC-15) was obtained from Sigma-Aldrich. Rabbit polyclonal anti-goat-HRP, anti-mouse HRP, and the polyclonal swine anti-rabbit-HRP were purchased from Dako.

### TLRs blockade with specific antibodies and receptor decoy

For TLRs blockade: cells were incubated with anti-TLR4, anti-TLR2 antibodies at the indicated concentration (cf. figure legends) during 1h at 37°C, and washed once with PBS. As a control, cells were incubated with mouse IgG2a isotype control antibodies. For TLR4 receptor decoy treatment: Tat protein was incubated with 10μg/ml of soluble recombinant human TLR4/MD2 protein during 1h at 37°C before being added into the wells. Tat protein solution incubated under the same conditions at 37°C was used as control.

### Methyl-β-cyclodextrin and dynasore treatments

Cells were plated in 24-well plates and pre-incubated for 60 minutes with various concentrations of M-βCD, a raft-disrupting drug, or for 30 min with various concentrations of dynasore for dynamin inhibition. As a control, cells were treated with similar amounts of dimethyl sulfoxide (DMSO) to those used to solubilize the inhibitors. After the drugs pretreatment, cells were incubated with Tat proteins (100 nM). As positive and negative controls, cells were stimulated with LPS or GST respectively. Eventual cytotoxic effects of the inhibitors were analyzed by the blue trypan dye exclusion assay. No related cytotoxicity was observed and cell viability was > 95% at the concentrations used.

### Cytokine detection by ELISA

Adherent human monocytes (10^6^/well), murine macrophages (5.10^5^/well) or HEK cells (5.10^5^/well) were washed 3 times with PBS. HEK cells were then cultured in the presence of 1% FCS, murine macrophages were cultured in the presence of 2% FCS. After 24 h of cell treatment, the supernatants were collected and analyzed for human and mouse IL-6 and IL-8. MoDCs and primary BDCA1+ DCs were plated at 10^6^/ml and kept in RPMI 10% FCS medium. Cytokine amounts were determined using ELISA kits from eBiosciences (for quantification of human IL-6 and mouse IL-6), R&D Systems (for quantification of human IL-8 and mouse CXCL1/KC) according to the manufacturers’ instructions.

### Cells transfection and Reporter assays

HEK cells were seeded into 24-well plates at 4.10^5^ cells the day before transfection. The cells (60–70% confluence) were co-transfected with indicated amounts of the NF-κB reporter plasmid (p-Nifty2-Seap, Invivogen) together with pORF^_^LacZ (Invivogen) using calcium phosphate transfection. After 24h of transfection, NF^_^κB driven SEAP-reporter gene expression was assayed in the supernatant as according to the manufacturer’s instructions (Invivogen). Data were background subtracted from transfected inactivated control wells and transfected with the corresponding empty vectors as controls. For normalization, cells were lysed and expression of β-galactosidase gene was analyzed.

### Isolation of cytoplasmic and nuclear proteins

Isolation of cytoplasmic, nuclear and membranes proteins extract were performed as previously described [41,61,63,64]. Briefly, for cytoplasmic and nuclear extraction, cells previously stimulated, were lysed at 4°C with 200 μL of hypotonic buffer A (Hepes 10 mM pH 7.9, KCl 10 mM, EDTA 0.1 mM, EGTA 0.1 mM, DTT 1 mM, PMSF 0.5 mM, Na3VO4 0.2 mM, NaF 0.05 mM) for 15 min. After addition of 12.5 μl of Nonidet P40 10% the lysate was strongly vortexed. After 1 min, 15366 g, 4°C the supernatant corresponding to the cytoplasm was collected. The nucleus pellets were solubilized in 100 μl of buffer B (Hepes 20 mM, pH 7.9, NaCl 0.4 M, EDTA 1 mM, EGTA 1 mM, DTT 1 mM, PMSF 1 mM, Na3VO4 0.2 mM, NaF 0.05 mM). Protein extracts were quantified by Bradford assay and stored at -20°C.

### Flow cytometry

HEK cells, pretreated or not for 30 min with dynasore (100 μM), were left non-stimulated or stimulated with GST-Tat 1–45, GST or LPS in complete medium containing 10% FCS, for the indicated time periods. Cells were then detached by cold PBS-EDTA 5 mM, washed with PBS-FCS 5%-Azide 0.01% and stained with anti-TLR4 (10 μg/ml, clone HTA125), anti-TLR4-PE or isotype control IgG2a-PE for 45 min on ice. After 3 washes, cells were incubated with secondary rabbit anti-mouse FITC-IgG (Becton Dickinson) for 45 min on ice. After washes, cells were fixed with PBS-1% PFA and analyzed by flow cytometry on a FACSCalibur (Becton, Dickinson). For intracellular cytokines staining cells were fixed and permeabilized during 20 min on ice with Cytofix/Cytoperm solution for BD Biosciences, then washed once with Perm/Wash solution (BD Biosciences), incubated with anti-IL-6-FITC and anti-IL-8-PE for 40 min on ice, washed twice with Perm/Wash buffer, and resuspended in PBS before analysis on Fortessa (BD).

### Statistical analyses

Statistical analysis was performed using GraphPad Prism software. All results are expressed as means +/- SD. All experiments were performed a minimum of three times. Differences in the means for the different groups was tested using one-way ANOVA followed by Bonferroni post hoc or paired Student's t test. A P-values < 0.05 was considered statistically significant. Statistical significance comparing "untreated" group versus "Treated (as indicated) " group are denoted with * for p < 0.05, ** p < 0.01, *** p < 0.001, ns non significant. Statistical significance comparing different group in dose response or inhibition experiments are linked with a black line above the compared bar and are denoted with # for p < 0.05, ## p < 0.01, ### p < 0.001, ns non significant.

## Results

### Tat protein induces production of IL-6 and IL-8 inflammatory cytokines by primary human monocytes

**Fig 1:**
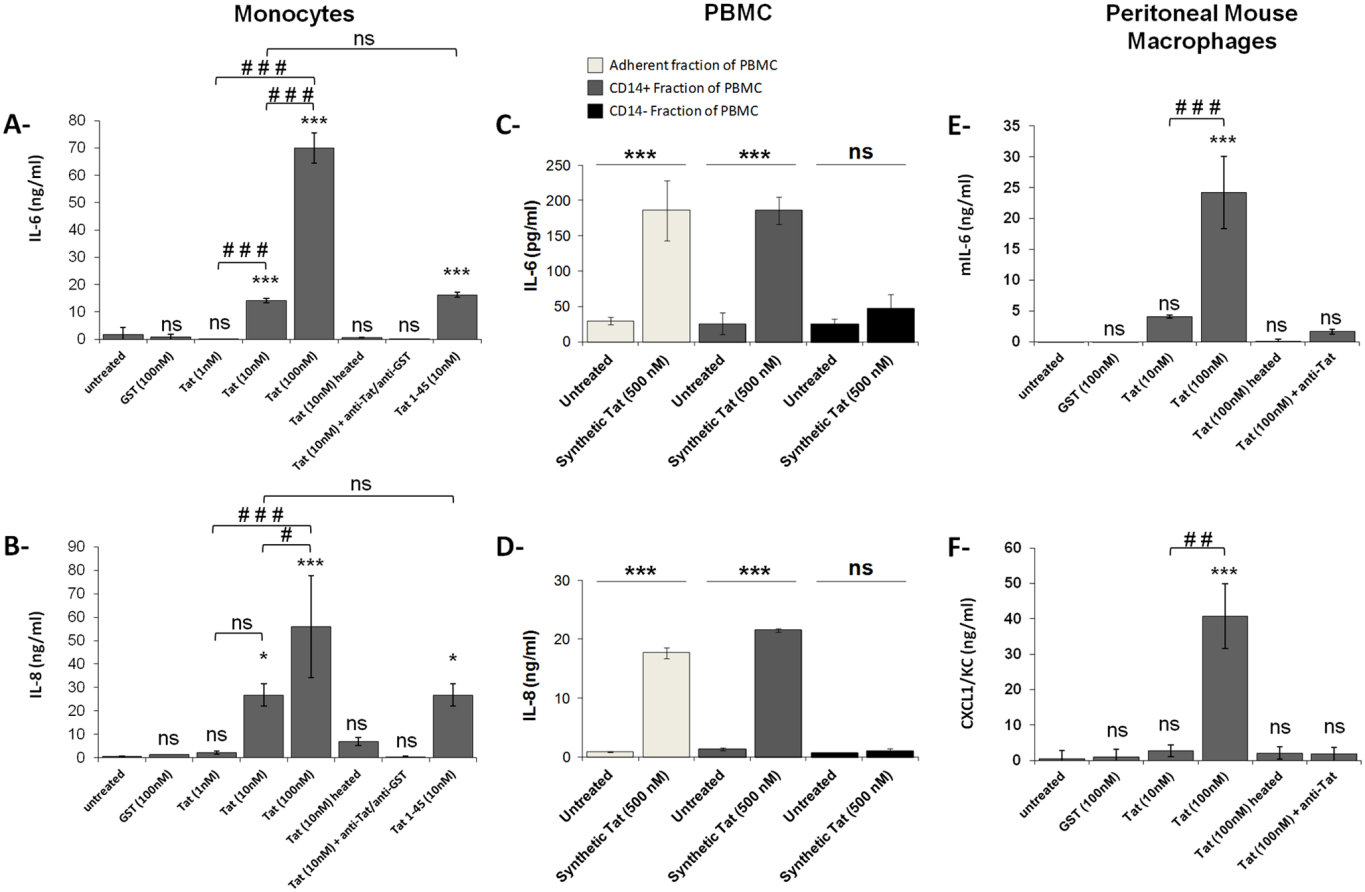
HIV-1 Tat induces IL-6 and IL-8 production in monocytes/macrophages. (**A-B**) and **(C-D)** Human monocytes (0.5x10^6^) or **(E-F)** Wt peritoneal mice macrophages (0.5x10^6^) were incubated with increasing amounts of recombinant GST-Tat 1–101 protein (Tat), a deleted mutant GST-Tat 1–45 protein carrying the first 45 amino acid (Tat 1–45) or an equal amount of GST protein alone (GST). Untreated cells were used as negative controls. Tat heat-inactivated for 20 min at 95°C or Tat previously incubated with mAb anti-Tat for 60 min at 37°C, or GST were used for the control of the specificity. **(C-D)** Monocytes were isolated from PBMC using either adherence protocol as described in Material and Methods, or positive selection using CD14 MicroBead according to the manufacturer instruction (Miltenyi Biotec), CD14 negative fraction of PBMC was used as control. Cells were either kept untreated or treated with Synthetic Tat. After 24 h of treatment, human or mouse IL-6 and human IL-8 and mouse CXCL1/KC cytokines were measured in the culture supernatants by ELISA. Cytokine production is expressed in ng/ml. The data represent means and standard deviation (SD) of three independent experiments. Asterisks represent *P* values comparing "untreated" group versus "Treated (as indicated)" group * for p < 0.05, ** p < 0.01, *** p < 0.001, ns non significant. Statistical significance comparing different group linked with a black line above the compared bar and are denoted with # for p < 0.05, ## p < 0.01, ### p < 0.001, ns non significant.

Because HIV-1 infection is associated with a progressive increase of pro-inflammatory cytokines, including IL-6 [67,68] and IL-8 [69,70], we set up a first experiment to assess whether HIV-1 Tat protein was involved in the production of these cytokines by primary monocytes isolated from either healthy volunteers or from HIV-1 infected patients. Monocytes isolated from healthy donors were left unstimulated (negative control), or were stimulated with a recombinant GST-Tat protein (1–100 nM) or with GST alone (control of specificity). The amount of cytokine produced in the culture supernatants, collected 24 h post-stimulation, was quantified using an ELISA assay. As shown in Fig 1A and 1B, HIV-1 Tat protein induced production of significant and dose-dependent amounts of IL-6 and IL-8 by monocytes. To demonstrate that these cytokines were produced by the monocytes fraction of PBMC and not by other cell types that could remain during the purification by adherence, we purified monocytes by positive selection that give rise to a purity of more than 99% of CD14+ monocytes and showed that only the CD14-positive cell fraction was able to produce IL-6 and IL-8 in response to Tat stimulation (S1A Fig).

These results were also confirmed, at least for IL-8, by the positive intracellular staining for IL-8 in CD14+ monocytes following Tat treatment (S1B Fig). The production of these cytokines was Tat-specific, since: (*i*) the production was abrogated in the presence of anti-Tat antibodies; (*ii*) no cytokines were detected in the supernatants of monocytes that were stimulated with heat-inactivated Tat protein; and (*iii*) no cytokine production was observed when stimulations were performed with GST alone (Fig 1A and 1B); iv) chemically synthesized HIV-1 Lai Tat protein is able to induce IL-6 and IL-8 in primary human monocyte isolated from PBMC by both adherence or positive selection protocols (Fig 1C and 1D) Similar results were obtained using peritoneal macrophages derived from wild type (Wt.) C57BL/6 mice (Fig 1E and 1F). These results indicate that the interaction of HIV-1 Tat protein with monocytes/macrophages stimulated production of IL-6 and IL-8 inflammatory cytokines. Although Tat stimulates the production of several additional cytokines including TNF-α, IL-10, IL-12, IFN-α and IFN-γ (data not shown), to investigate the implication of TLR4 and its downstream pathways, in this study we focused, as a model, on IL-6 and IL-8 production by primary cells and then IL-8 production in HEK-cell line.

### Engagement of TLR4 by Tat protein is required for IL-6 and IL-8 production by monocytes/macrophages

Structure-relationship studies using Tat-deleted mutants allowed us to show that N-terminal GST-Tat 1–45 was sufficient to stimulate TNF-α and IL-10 production in human monocytes [33,71]. In the present study, we showed that N-terminal GST-Tat 1–45 was sufficient to stimulate IL-6 and IL-8 production (Fig 1A and 1B). This result suggests that the 1–45 N-terminal Tat region, which lacks the basic Tat-domain essential for Tat uptake, mediated cytokine production by recruiting a potential receptor present at the cell surface.

We therefore hypothesized that TLR4 would be a receptor for Tat protein based on: (*i*) the above results; (*ii*) our recent indirect observations that Tat protein induced TNF-α and IL-10 cytokines by activating PKC, MAP-Kinases and NF-κB pathways [22,41,60–62,64,71] pathways that are recruited downstream of TLR4 engagement [72–74]; and (*iii*) another recent indirect observation that Tat-cell functional interaction was abrogated in the presence of anti-TLR4 antibodies [33]. To test this hypothesis, we used four direct complementary approaches to analyze the effect of: (*i*) soluble recombinant TLR4/MD2 as a receptor decoy; (*ii*) HEK cells response to Tat stimulation following stable transfection with TLR4-MD2-CD14; (*iii*) Tat on the modulation of TLR4 expression; and (*iv*) Tat in stimulating NF-κB pathway activation, which is considered as a signature of TLR4 engagement.

**Fig 2:**
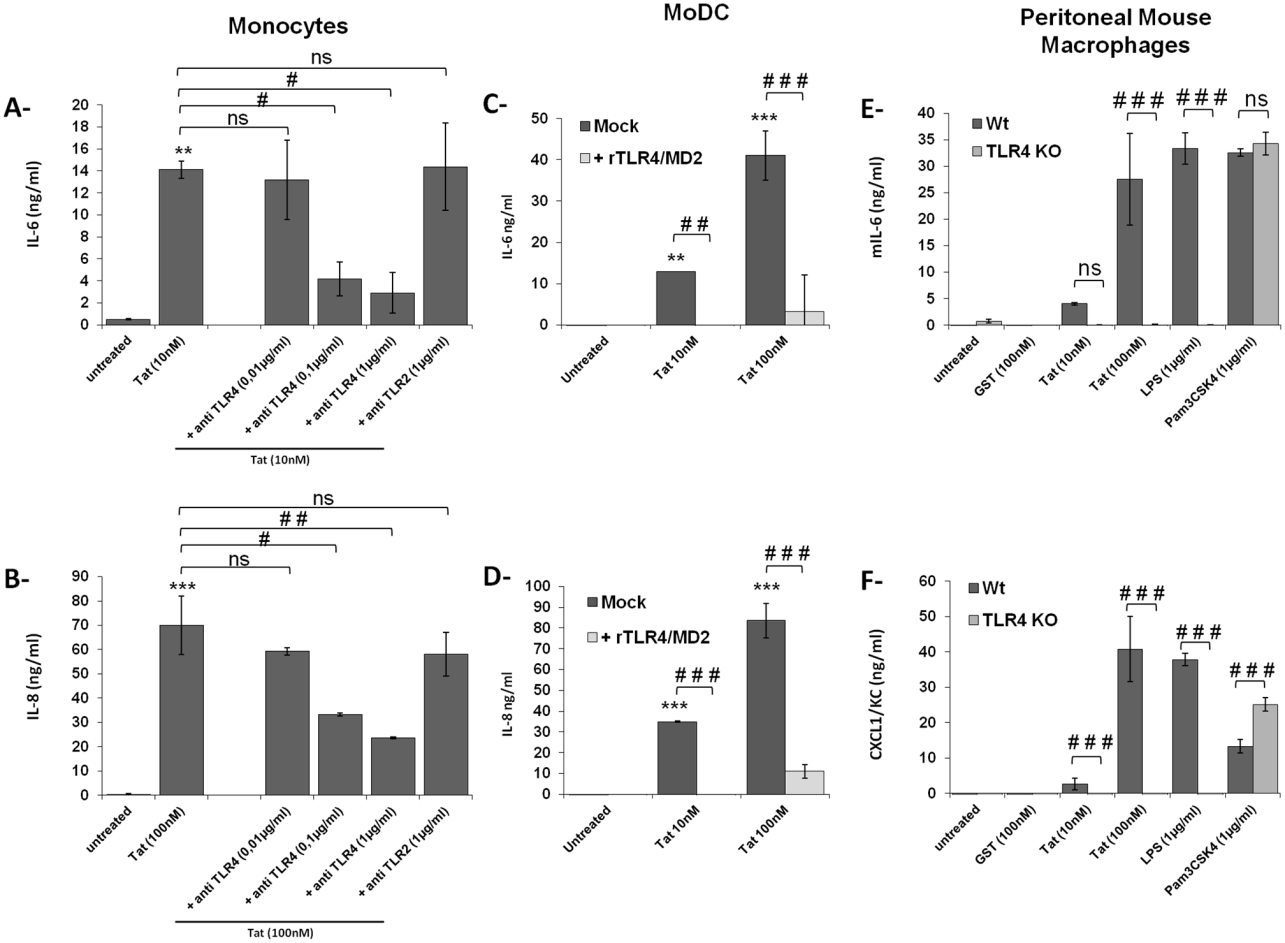
HIV-1 Tat induces IL-6 and IL-8 production in a TLR4-CD14-MD2 dependent manner. (**A-B**) Human monocytes were pretreated or not 1h, with increasing amounts of blocking antibodies against TLR4 (0.01–1μg/ml), or TLR2 (1μg/ml) before stimulation with GST-Tat 101 protein (10nM, 100nM). Culture supernatants were recovered after 24 h and IL-6 and IL-8 production was measured by ELISA. (**C-D**) Monocytes derived Dendritic Cells (MoDC) were treated with increasing amounts of GST-Tat 1–101 protein pre-incubated (light grey histograms) or not (dark grey histograms) with recombinant human TLR4/MD2 (10μg/ml) for 1h at 37°C. After 24 hrs., culture supernatants were recovered and IL-6 and IL-8 production was measured by ELISA. (**E-F**) Peritoneal macrophages from wild type (dark grey histograms) or TLR4 KO mice (light grey histograms) were stimulated for 24 h with increasing concentrations of GST-Tat 1–101 protein. Control experiments were performed by using the following TLR ligands: LPS (TLR4-CD14-MD2) and Pam3CSK4 (TLR2-CD14). Mouse IL-6 and CXCL1/KC production was determined by ELISA. The data represent means +/- SD of three independent experiments. Statistical significance comparing "untreated" group versus "Treated (as indicated)" group are denoted with * for p < 0.05, ** p < 0.01, *** p < 0.001, ns non significant. Statistical significance comparing different groups linked with a black line above the compared bar and are denoted with # for p < 0.05, ## p < 0.01, ### p < 0.001, ns non significant.

As a first approach, to determine whether TLR4 pathway is involved in Tat induced IL-6 and IL-8 cytokines, primary monocytes were left untreated (negative control) or stimulated with recombinant GST-Tat 1–101, with or without anti-TLR4 blocking antibodies. As a control of specificity, we used anti-TLR2 blocking antibodies, which recognized TLR2/TLR1 and TLR2/TLR6 heterodimers. Under these conditions, significant levels of IL-6 and IL-8 were detected in the supernatants of monocytes following GST-Tat 1–101 stimulation (Fig 2A and 2B). In contrast, the production of both IL-6 and IL-8 was strongly inhibited (more than 60%) in the presence of anti-TLR4 blocking antibodies, suggesting the involvement of TLR-4 in Tat-monocyte functional interaction (Fig 2A and 2B). In contrast, no inhibition of IL-6 or IL-8 production was observed when the same experiments were performed in the presence of anti-TLR2 antibodies instead of anti-TLR4 antibodies, confirming the specificity of Tat-TLR4 interaction (Fig 2A and 2B).

In a more direct approach, we investigated the effect of recombinant soluble TLR4 (rTLR4/MD2) as an antagonist. Under these conditions, a total inhibition of the capacity of Tat to induce production of IL-6 and IL-8 was observed in MoDCs (Fig 2C and 2D). This result demonstrates that soluble TLR4/MD2 acts as a decoy receptor, thus capturing soluble Tat protein, preventing its binding with TLR4-membrane receptor and, consequently, leading to total inhibition of IL-6 and IL-8 production.

To further analyze the implication of TLR4 in the production of IL-6 and CXCL1/CK (a functional homologue of human IL-8) cytokines/chemokines following Tat stimulation in mouse model, peritoneal macrophages isolated from Wt. or from TLR4 KO mice were stimulated with increasing amounts of Tat protein. The amount of IL-6 and CXCL1/KC produced in the supernatants was measured by ELISA 24 h after stimulation. As shown in Fig 2E and 2F, Tat induced production of both IL-6 and CXCL1/KC, in a dose-dependent manner, by peritoneal macrophages from Wt mice. In contrast, no significant level of IL-6 and IL-8 cytokines was produced by macrophages from TLR4 KO mice. As controls, TLR4 KO mice continued to respond to the stimulation with TLR2 ligand, Pam3CSK4 (Fig 2E and 2F).

Altogether, these results confirm that a direct engagement of TLR4 by Tat protein is required for IL-6 and IL-8 production.

**Fig 3:**
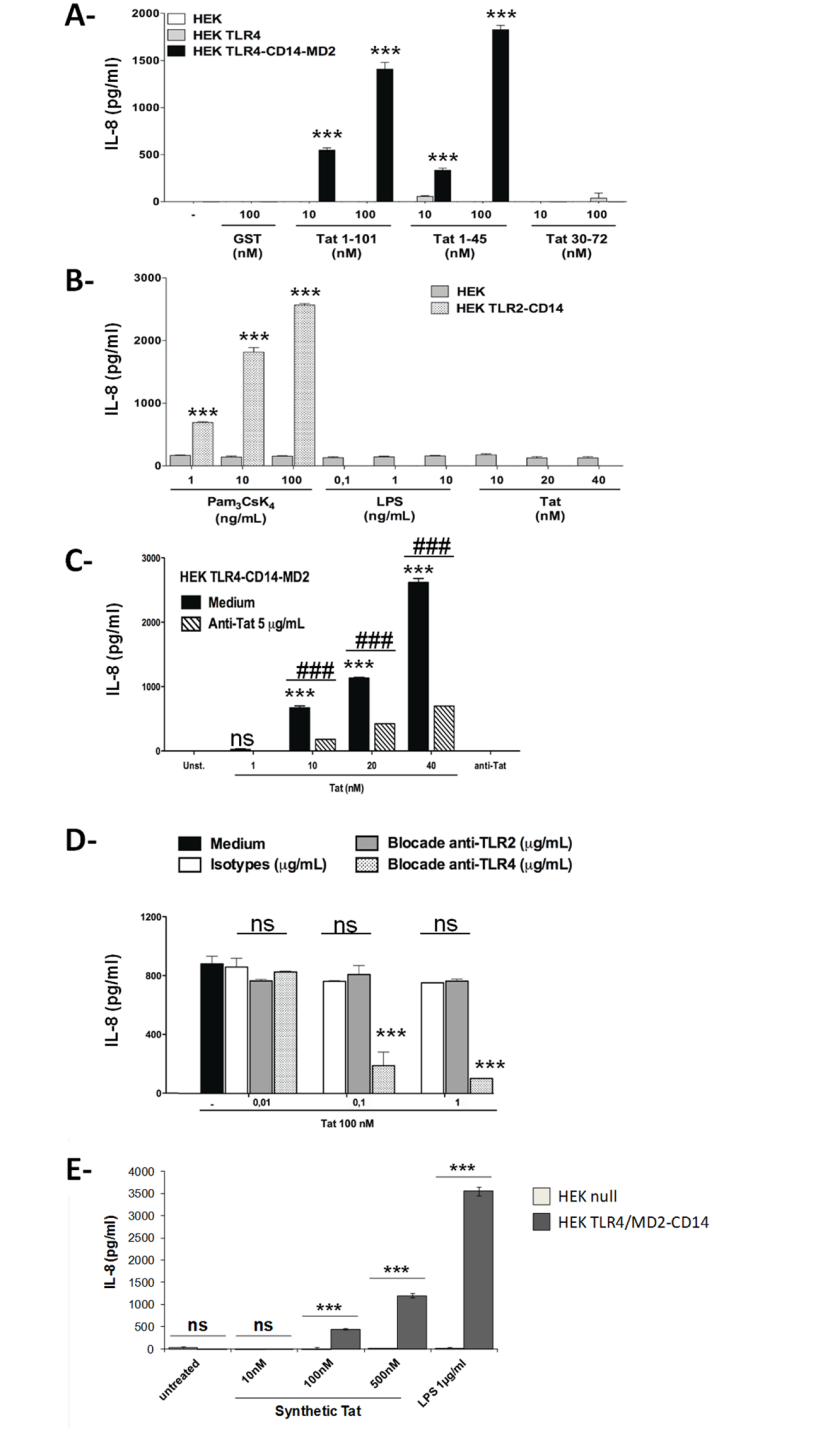
HIV-1 Tat induces production of IL-8 production in a TLR4-CD14-MD2 dependent manner. (**A**) HEK cell lines expressing TLR4 (grey histograms), TLR4-CD14-MD2 (black histograms), or HEK null, carrying an empty plasmid (white histograms) were pretreated or not with 10 and 100 nM of GST-Tat 1–101 or its deleted mutants 1–45 or 30–72. After 24 hrs., culture supernatants were recovered and IL-8 production was measured by ELISA. The data represent means +/- SD of three independent experiments. (**B**) HEK Null or HEK cell expressing TLR2-CD14 were pretreated or not with indicated amounts of GST-Tat 1–101 or LPS or Pam3CSK4. After 24h, IL-8 production in culture supernatant was analyzed by ELISA. The data represent means +/- SD of three independent experiments. (**C**) HEK-TLR4-CD14-MD2 cells were stimulated with Tat protein (Black histograms) or Tat protein previously incubated with 5 μg/mL of anti-Tat blocking antibodies (hatched histograms). After 24h of stimulation, IL-8 production induced by Tat was quantified in the culture supernatant by ELISA. The data represent means +/- SD of three independent experiments. (**D**) HEK-TLR4-CD14-MD2 cells were previously treated with increasing amounts of mAb anti-TLR4 or anti-TLR2 or isotypes control for 60 min before stimulation with Tat. After 24h, IL-8 production was measured in the culture supernatants by ELISA. The results are expressed in pg/ml. (E) HEK cell lines stably expressing TLR4/MD2-CD14 (HEK TLR4/MD2-CD14) or an empty plasmid (HEK Null) from InvivoGen were either kept untreated or treated with escalating doses of Synthetic Tat, or LPS as a positive control. After 24h of incubation, cell supernatant was collected and used for cytokine quantification by ELISA as described in Materials and Methods. The values are representative of at least three independent experiments. Statistical significance comparing "untreated" group versus "Treated (as indicated) " group are denoted with * for p < 0.05, ** p < 0.01, *** p < 0.001, ns non significant. Statistical significance comparing different group linked with a black line above the compared bar and are denoted with # for p < 0.05, ## p < 0.01, ### p < 0.001, ns non significant.

We next analyzed the implication of TLR4 and its CD14 and MD2 cofactors in Tat-induced cytokine production, at a molecular level. We used human embryonic kidney HEK293 cells stably expressing TLR4, either alone (HEK TLR4) or in association with its cofactors, CD14 and MD2 (HEK TLR4/MD2-CD14). HEK cells transfected with an empty plasmid (HEK Null) and HEK transfected with TLR2 and CD14 (HEK TLR2-CD14) were used as controls. HEK cells were stimulated with increasing doses of GST-Tat 1–101, GST-Tat 1–45 or GST-Tat 30–72. Since, these HEK cell lines are not seem endowed with the capacity to produce IL-6 (according to the supplier InvivoGen and our observation) only the amount of IL-8 produced in the supernatants was measured by ELISA 24 h after stimulation. As shown in Fig 3A, both Tat 1–101 and Tat 1–45 induced a significant and dose-dependent amount of IL-8 in HEK cells expressing TLR4, CD14 and MD2 cofactors, but not in HEK-TLR4 and HEK-Null confirming the results presented above using human monocytes and mouse macrophages (Figs 1 and 2). In contrast, no cytokine production was detected in cell supernatants from HEK cells transfected with TLR4 alone or TLR2-CD14 or from HEK-Null cells (Fig 3A and 3B). Moreover, no cytokine production was observed with the deleted mutant GST-Tat 30–72 or GST (Fig 3A). This result confirms that the 1–45 N-terminal Tat domain, which lacks the basic Tat-domain essential for Tat-uptake, mediated cytokine production by recruiting TLR4 at the cell surface. As shown in Fig 3C, the addition of anti-Tat antibodies abrogated IL-8 production, further confirming the activation of TLR4 pathway by HIV-1 Tat protein. Moreover, the production IL-8 induced by Tat in HEK TLR4/MD2-CD14 cell line was inhibited in the presence of increasing amounts of blocking anti-TLR4 mAbs, thus further confirming the activation of TLR4 pathway in this system (Fig 3D). A complete inhibition was obtained with anti-TLR4 mAbs at a dose as low as 1 μg/ml. In line with these results we also show that chemically synthesized HIV-1 Tat protein is able to induce IL-8 production in HEK cell lines in a TLR4/MD2, CD14 dependent mechanism (Fig 3E). As controls, we showed that: (*i*) both anti-TLR2 mAbs and isotype control mAbs had no effect on the production of IL-8 cytokine by HEK-TLR4-CD14-MD2 cells stimulated by Tat (Fig 3D); and, (*ii*) Pam3CSK4-stimulated HEK-TLR2-CD14 (Fig 3B) and LPS-stimulated HEK-TLR4-CD14-MD2 continued to produce IL-8 (data not shown). Collectively, the above results demonstrate that Tat protein recruits TLR4-CD14-MD2 receptor, via its 1–45 N-terminal domain, for the production of the IL-6 and IL-8 inflammatory cytokines.

### Tat protein induces production of IL-6 and IL-8 by monocytes and dendritic cells derived from both healthy individuals and HIV-1 infected patients

**Fig 4:**
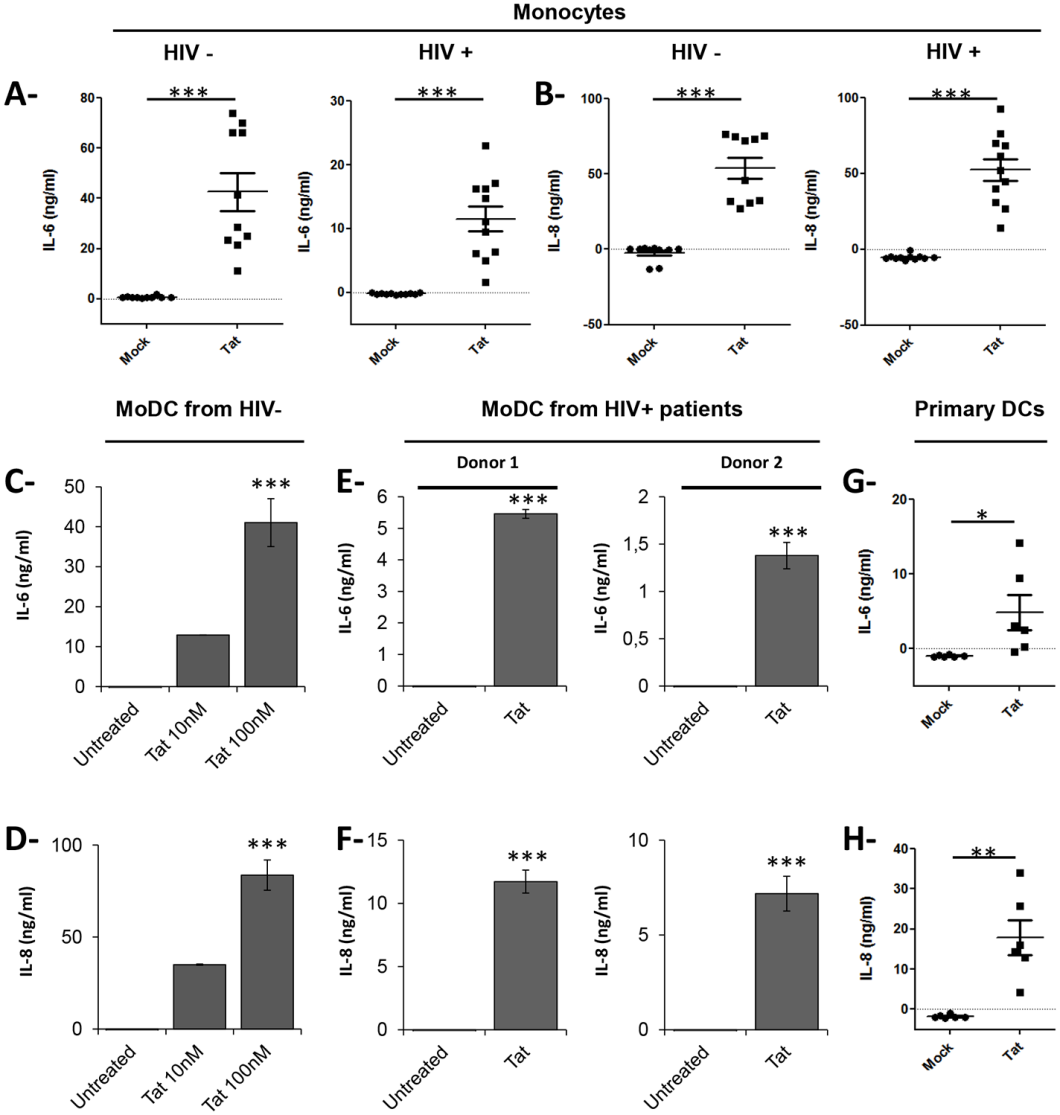
HIV-1 Tat Protein stimulates the production of IL-6 and IL-8 in monocytes and dendritic cells from HIV-1 infected and uninfected patients. (**A-B**): Monocytes from HIV negative donors (n = 10) or HIV-1 infected patients (n = 11) were treated with GST-Tat 1–101 protein (100nM), or not (Mock). After 24 h of treatment, IL-6 (**A**) and IL-8 (**B**) were quantified in the cell supernatants. (**C-F)** MoDCs from healthy donors (n = 3) or from two HIV-1 infected donors were treated with GST-Tat 1–101 protein (100nM), or not (Untreated). After 24 h of treatment, IL-6 (**C, E**) and IL-8 (**D, F**) were quantified in the cell supernatants. (**G-H**): Primary myeloid dendritic cells (CD1c + subset) were treated with Tat protein (100nM), or not (Mock). After 24 h of treatment, IL-6 (**G**) and IL-8 (**H**) were quantified in the cell supernatants. Differences in the means for the different groups were tested using Student's t test. Asterisks represent *P* values: *, *P <* 0.05; **, *P ˂* 0.01; ***, *P ˂* 0.001.

Dendritic cells (DCs) play a crucial role in the induction of a protective innate and adaptive immunity against viral infections, including HIV-1 [75]. Therefore, we next investigated whether human DCs would produce IL-6 and IL-8 following stimulation with HIV-1 Tat protein. MoDCs obtained from either healthy subjects or from HIV-1 infected patients were treated or not with Tat protein and secreted or intracellular IL-6 and IL-8 were analyzed. In these experiments, primary human monocytes isolated from the same individuals were used in parallel, as controls. Similar to primary human monocytes (Fig 4A and 4B), MoDC derived from healthy donors (HIV-) also produced significant, dose-dependent, amounts of IL-6 and IL-8 inflammatory cytokines when stimulated with HIV-1 Tat protein (*p* < 0.005 Fig 4C and 4D). More interestingly, both primary human monocytes and MoDCs obtained from HIV-1 infected patients produced significant amounts of IL-6 and IL-8 in response to HIV-1 Tat protein (Fig 4E to 4F). In parallel, the analysis of intracellular staining for IL-6 and IL-8 in Tat-untreated PBMCs collected from HIV-1 infected patients with detectable viral load, showed the intracellular presence of IL-8 in both CD14 and CD3-positive cells (S2A and S2B Fig). However the frequency of CD14+ cells positives for IL-8 (4,4% to 11,8%) is greater than that obtained with CD3+ cells (0,17 to 1,1%) (S2A and S2B Fig). In contrast, no significant positive intracellular IL-6 staining was detected both in CD14+ and CD3+ cells of the same HIV-1 positive patients (S2A and S2B Fig). No intracellular IL-6 and IL-8 staining was detected both in CD14+ and CD3+ cells of healthy donors (S2C Fig). These results suggest that during HIV-1 infection, primary myeloid cells, such as MoDC and monocyte)s, produce IL-6 and IL-8 pro-inflammatory cytokine when stimulated with HIV-1 Tat.

A mechanism mediated by pro-inflammatory cytokines, such as IL6 and IL-8, would contribute to immune dysregulation seen in HIV-1 infected patients and, hence, promoting HIV-1 viral replication and progression to AIDS.

Since monocyte-derived dendritic cells were used in the above experiments as a model of myeloid DC, we wondered whether human primary myeloid DCs will similarly produce IL-6 and IL-8 inflammatory cytokines when stimulated with HIV-1 Tat. Because only few primary myeloid DCs are in the blood and to avoid their activation during positive sorting, primary myeloid DCs were instead isolated by negative selection from PBMCs of healthy donors (HIV-). Negative selected primary myeloid DCs were then tested for their capacity to produce IL-6 and IL-8 cytokines following stimulation by HIV-1 Tat protein, as above. As shown in Fig 4G and 4H, HIV-1 Tat protein stimulated the production of significant amounts of both IL-6 and IL-8 inflammatory cytokines by primary human myeloid DCs (Fig 4G and 4H). For practical reasons, due to the rarity of primary myeloid DCs populations in the blood of HIV-1 infected patients, Tat stimulation of primary DCs from HIV-1 infected patients could not be performed.

### Tat protein activates NF-κB pathway in a TLR4-CD14-MD2-dependent manner

Considering that engagement of all TLR pathways lead to the activation of NF-κB transcription factor [74], we assessed whether HIV-1 Tat protein would also activate NF-κB transcription factor. In inactivated cells, NF-κB remains sequestered in the cytoplasm by its inhibitor IκB. In contrast, in activated cells, IκB undergoes phosphorylation leading to its dissociation from NF-κB. Free NF-κB can then be translocated to the nucleus where it binds to its specific sites. Thus, in order to study the effect of Tat protein on the activation of NF-κB and the role of this activation on IL-6 and IL-8 production, several complementary experiments were performed.

**Fig 5:**
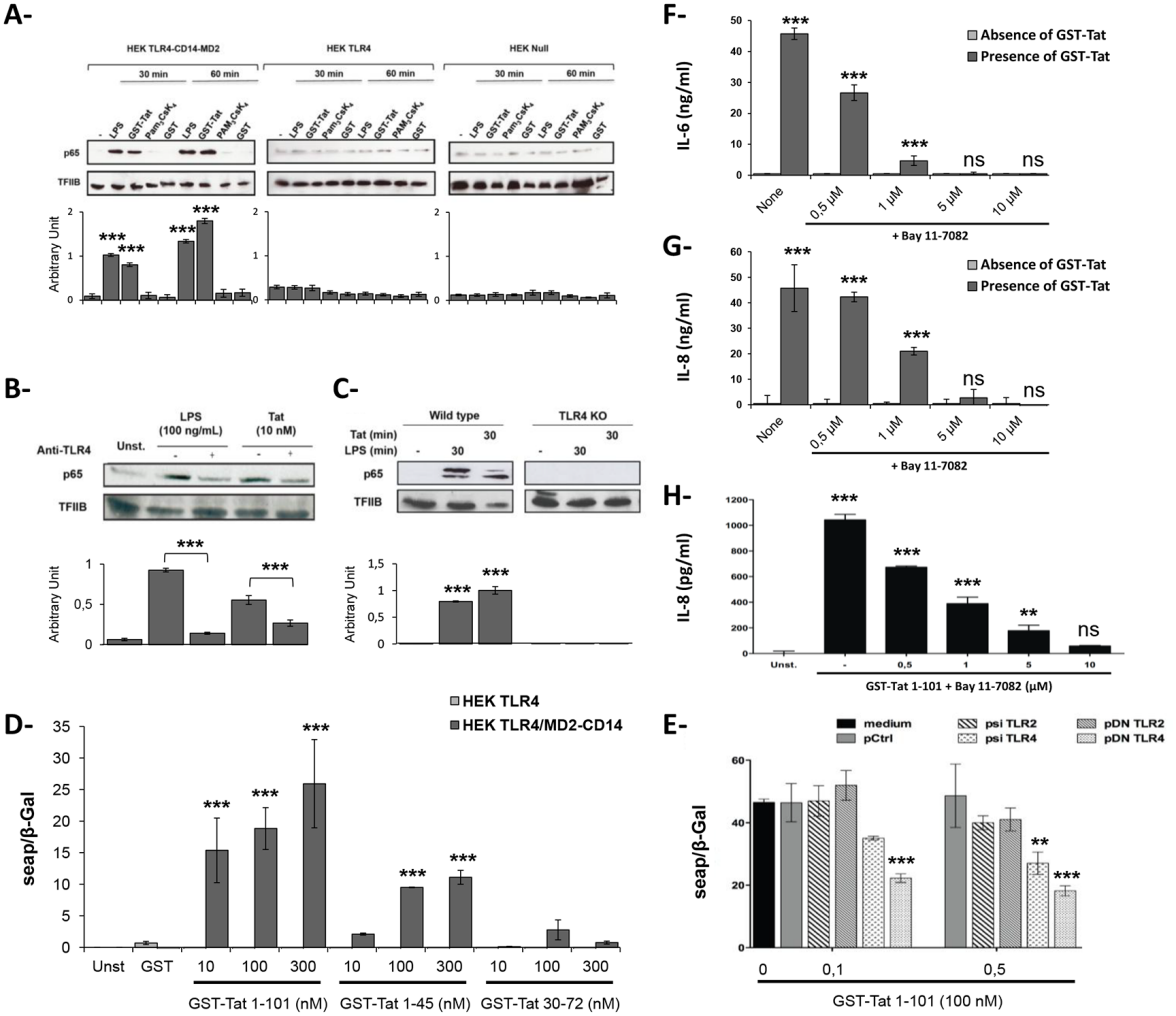
HIV-1 Tat activates NF-κB pathway ina TLR4 dependent manner. (**A**) HEK null, HEK-TLR4, HEK-TLR4-CD14-MD2, (**B**) primary human monocytes, pretreated or not with anti-TLR4 (1μg/ml) or, (**C**) peritoneal macrophages from wt or TLR4 KO mice cells were stimulated during 30 or 60 minutes with total Tat protein (10 nM). Similar stimulations were also conducted with GST, or Pam3CSK or LPS TLR ligands. p65 subunit of NF-κB was detected by Western-Blot in the nucleic fraction of the cells. Quantification of the band obtained from 3 independent experiment was performed using Image J Software. (**D-E**) HEK TLR4 or HEK expressing TLR4-CD14-MD2 cell lines were co-transfected with same amounts of the NF-κB reporter plasmid together with pORF-LacZ and then stimulated with increasing amounts of GST-Tat 1–101, GST-Tat 1–45, GST-Tat 30–72 or GST alone as negative control (**D**). In (**E**), cells were also co-transfected with the indicated amounts of the plasmid encoding siRNA or Dominant Negative (control, TLR4 or TLR2). After 24h, cells were stimulated with total Tat or its deleted mutants. After 24h of stimulation, NF-κB driven SEAP-reporter gene expression was measured in the culture supernatants. For normalization, cells were lysed and expression of β-galactosidase gene was analyzed. (**F-H**) IL-6 and IL-8 cytokine production was analyzed in the culture supernatants of HEK-TLR4-MD2-CD14 cells (**F**) and human monocytes (**G-H**) previously treated with non-toxic concentrations of Bay11-7082 (0.5–10μM). Asterisks represent *P* values: *, *P ˂* 0.05; **, *P ˂* 0.01; ***, *P ˂* 0.001, ns non significant.

In a first experiment, we showed that Tat protein treatment of HEK cell lines that were stably transfected with TLR4-MD2-CD14 induced the nuclear translocation of NF-κB as demonstrated by the presence of p65 protein, the RelA subunit of NF-κB, in the nuclear fraction of Tat-stimulated cells Fig 5A. In contrast, nuclear translocation of NF-κB was not observed in similar experiments using HEK-TLR4 or HEK-Null cells, thus suggesting the importance of the integrity of TLR4-MD2-CD14 complex expression in ensuring the signaling transduction. As a positive control, LPS induced activation of NF-κB in HEK TLR4-MD2-CD14 cells (Fig 5A). As expected, nuclear translocation of NF-κB was not detected in non-stimulated HEK cells (negative control) (Fig 5A–5C). In addition, Tat protein was also able to activate NF-κB in primary human monocytes as well as in mouse peritoneal macrophages (Fig 5B and 5C). Interestingly, we found that NF-κB activation: (*i*) was totally abolished in Tat-treated peritoneal macrophages obtained from TLR4 KO mice (Fig 5C); and (*ii*) was strongly inhibited in primary human monocytes stimulated with Tat, in the presence of blocking anti-TLR4 mAbs (Fig 5B).

In a second experiment, the capacity of Tat protein to activate NF-κB following the activation of the TLR4-MD2-CD14 pathway was further evaluated in a HEK-cell line stably expressing TLR4 or TLR4-MD2-CD14 complex and transfected with pNifty2-seap, a plasmid harboring a gene coding for a secreted alkaline phosphatase (SEAP) under the control of NF-κB. These cells were then treated with escalating doses of HIV-1 Tat mutants for 24h and SEAP activity was quantified in cell supernatants, as described in the *Materials & Methods*. As shown in Fig 5D, both GST-Tat 1–101 and GST-Tat 1–45 stimulated significant levels of SEAP expression in a dose-dependent manner but GST-Tat 30–72 or GST alone did not. Most importantly, Tat-induced SEAP expression was observed in HEK-TLR4-MD2-CD14 cells, but was absent in HEK-TLR4 cells (Fig 5D) and in HEK-Null (*data not shown*). This result indicates that NF-κB activation by HIV-1 Tat and by its 1–45 N-terminal domain is tightly associated with TLR4-MD2-CD14 complex expression. To further support this assessment, similar experiments were performed using cells pre-transfected with psi-TLR2, psi-TLR4, pDN-TLR2 or pDN-TLR4 plasmid vectors expressing si-RNA or negative dominant plasmids selectively targeting TLR2 or TLR4. Under these conditions, significant inhibitions of Tat-induced SEAP were obtained in the presence of psi-TLR4 (40%) and pDN-TLR4 plasmids (60%) (Fig 5E). No inhibition or only small effects were observed with psi-TLR2 and pDN-TLR2 plasmids or with pCtrl vehicle alone used as controls, (Fig 5E).

Finally, the involvement of NF-κB pathway in Tat-induced IL-6 and IL-8 cytokines was confirmed using a specific inhibitor (Bay11-7082). Primary human monocytes and HEK-TLR4-MD2-CD14 cells were first treated with non-toxic doses (0.5–10 μM) of Bay11-7082 inhibitor, then stimulated with HIV-1 Tat protein, as above. Under these conditions, the production of both IL-6 and IL-8 cytokines was significantly inhibited, in a dose-dependent manner, both in primary human monocytes (Fig 5F and 5G) and in HEK-TLR4-MD4-CD14 cells (Fig 5H). Altogether, the above results demonstrate that HIV-1 Tat protein stimulated the production of IL-6 and IL-8 pro-inflammatory cytokines in primary human monocytes by recruiting the TLR4-MD2-CD14 complex followed by stimulation of the NF-κB pathway.

### HIV-1 Tat protein-TLR4 interaction leads to TLR4 down-regulation and SOCS1 activation

**Fig 6:**
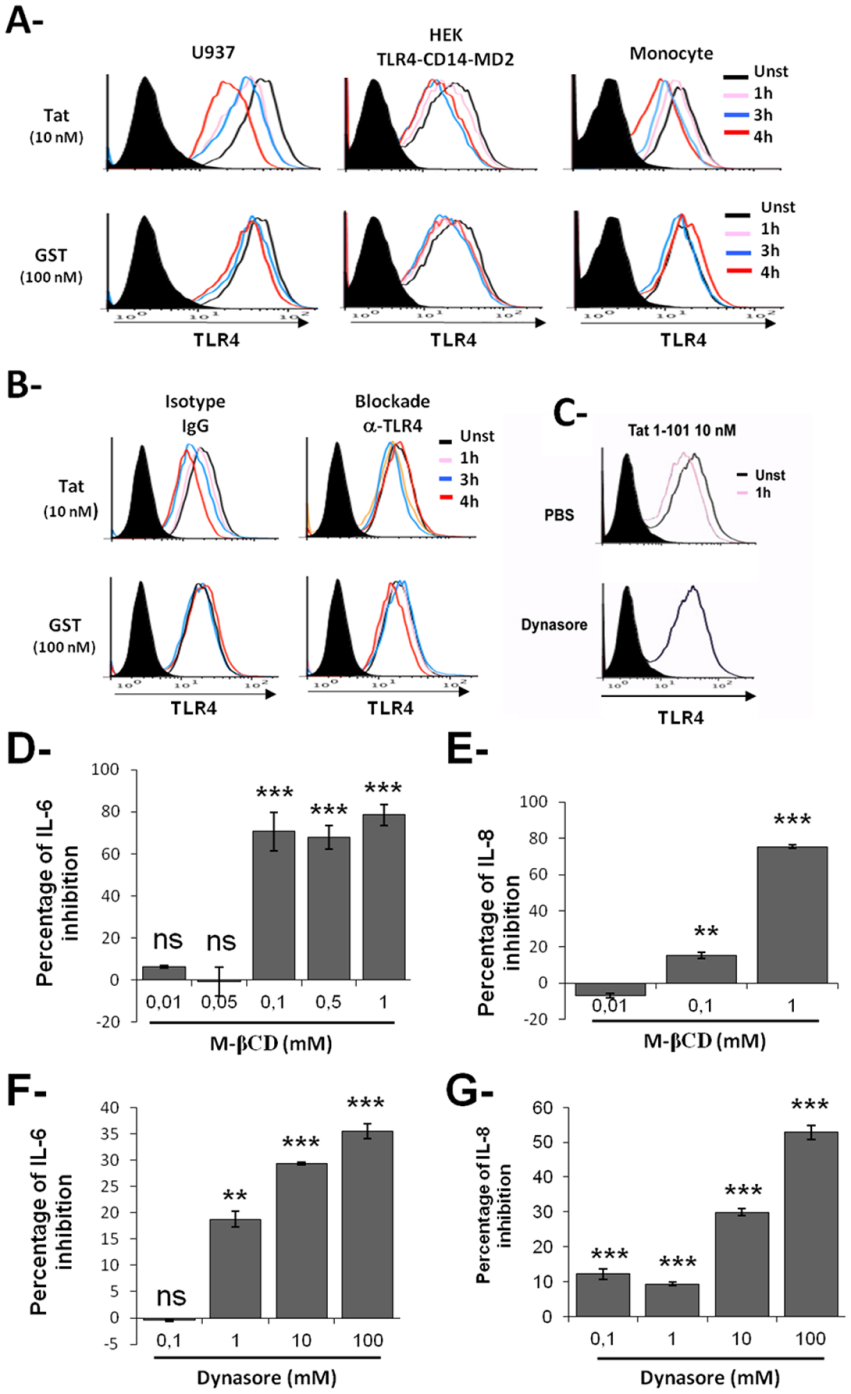
Tat induces TLR4 down modulation by specific dynamin endocytosis. (**A**) U937, HEK-TLR4-MD2-CD14 or human monocytes were stimulated or not with 10 or 100 nM of GST +/-Tat for the indicated times. Surface expression of TLR4 in unstimulated (black line) or stimulated (pink, blue and red lines) cells was analyzed by flow cytometry using anti-TLR4 or isotype control IgG (tinted), stained with secondary FITC or PE antibodies. Data represent one of three independent experiments. (**B**) U937 were pre-incubated for 1 h with 1 μg/mL of blocking anti-TLR4 or (**C**) HEK TLR4-CD14-MD2 were pretreated with 80 μM of dynasore for 30 minutes before stimulation with GST+/-Tat (100 nM) for the time periods indicated. Cell surface expression of TLR4 was then analyzed as described above. (**D**–**G**) The integrity of the raft structure is necessary for Tat-induced IL-6 and IL-8: **D-E**) Purified human monocytes (10^6^) were pretreated with increasing amounts of raft disruption drug M-β-CD (10, 60 min) or **F-G**) with dynasore for 30 minutes before stimulation with GST-Tat 1–101 (100 nM). After 24 h, IL-6 and IL-8 production in the culture supernatants were quantified by ELISA. The data represent means (pg/ml) and SD (n >3). As controls, cells were stimulated with PBS, DMSO or the highest drug concentration and cytokine production and cytotoxicity (trypan blue) were analyzed. Asterisks represent *P* values: *, *P ˂* 0.05; **, *P ˂* 0.01; ***, *P ˂* 0.001, ns non significant.

We next studied, how TLR4 activation by Tat could be negatively regulated. To this end we investigated the effect of HIV-1 Tat protein on the expression of TLR4 and of SOCS1, a protein associated with HIV-1 infection and disease progression [76,77]. The promonocytic U937, HEK TLR4-CD14-MD2 cell lines, and primary human monocytes were treated for 1, 3 or 4 hrs. with either Tat protein or with GST alone (control). The modulation of cell surface TLR4 was then analyzed by flow cytometry using a mAbs specific to TLR4. As shown in Fig 6A, treatment with Tat protein, but not GST, induced a time-dependent down-regulation of TLR4 on the surface of U937 and HEK-TLR4-MD2-CD14 cell lines as well as on primary human monocytes. The down-regulation of TLR4 was detected as early as 1 hr. post-treatment with Tat (Fig 6A). Detection of TLR4 at the cell surface by anti-TLR4-PE antibodies was totally abolished following cells pre-treatment with saturating concentrations of non PE-labeled anti-TLR4 blocking mAbs, indicating that the Tat effect on TLR4 internalization was specific (Fig 6B). Furthermore, pre-treatment of cells with dynasore, a dynamin-dependent endosomal scission inhibitor, completely prevented Tat-TLR4 internalization (Fig 6C), thus confirming the specificity of the Tat-TLR4 down modulation and excluding a masking effect by Tat. One can note that blockade of TLR4 internalization by dynasore led to a slight inhibition of IL-8 and IL-6 production (Fig 6F and 6G). These results strongly suggest that Tat-mediated internalization of TLR4 is dynamin-dependent and that expression of a sufficient amount of TLR4 on the cell surface is essential for efficient induction pro-inflammatory cytokines by HIV-1 Tat suggesting the implication of both Myd88 and Trif TLR4 pathways.

To support the above result, we analyzed whether the integrity of the plasma membrane would affect TLR4 cell surface expression and IL-6 and IL-8 production induced by Tat protein. Human monocytes were pre-treated with methyl-β-cyclodextrin (M-βCD), a compound that selectively depletes cholesterol from the plasma membrane, and cell surface expression of TLR4 and production of cytokine were determined. Treatment with M-βCD, for 10 and 60 min, had no significant effect on TLR4 expression but it significantly reduced the amount of IL-8 and IL-6 produced (Fig 6D and 6E). These results suggest that while lipidic raft structures did not appear to affect cell surface expression of TLR4, its integrity remains an important factor for efficient stimulation of the TLR4-MD2 signaling pathway that leads to the production of pro-inflammatory cytokines.

**Fig 7:**
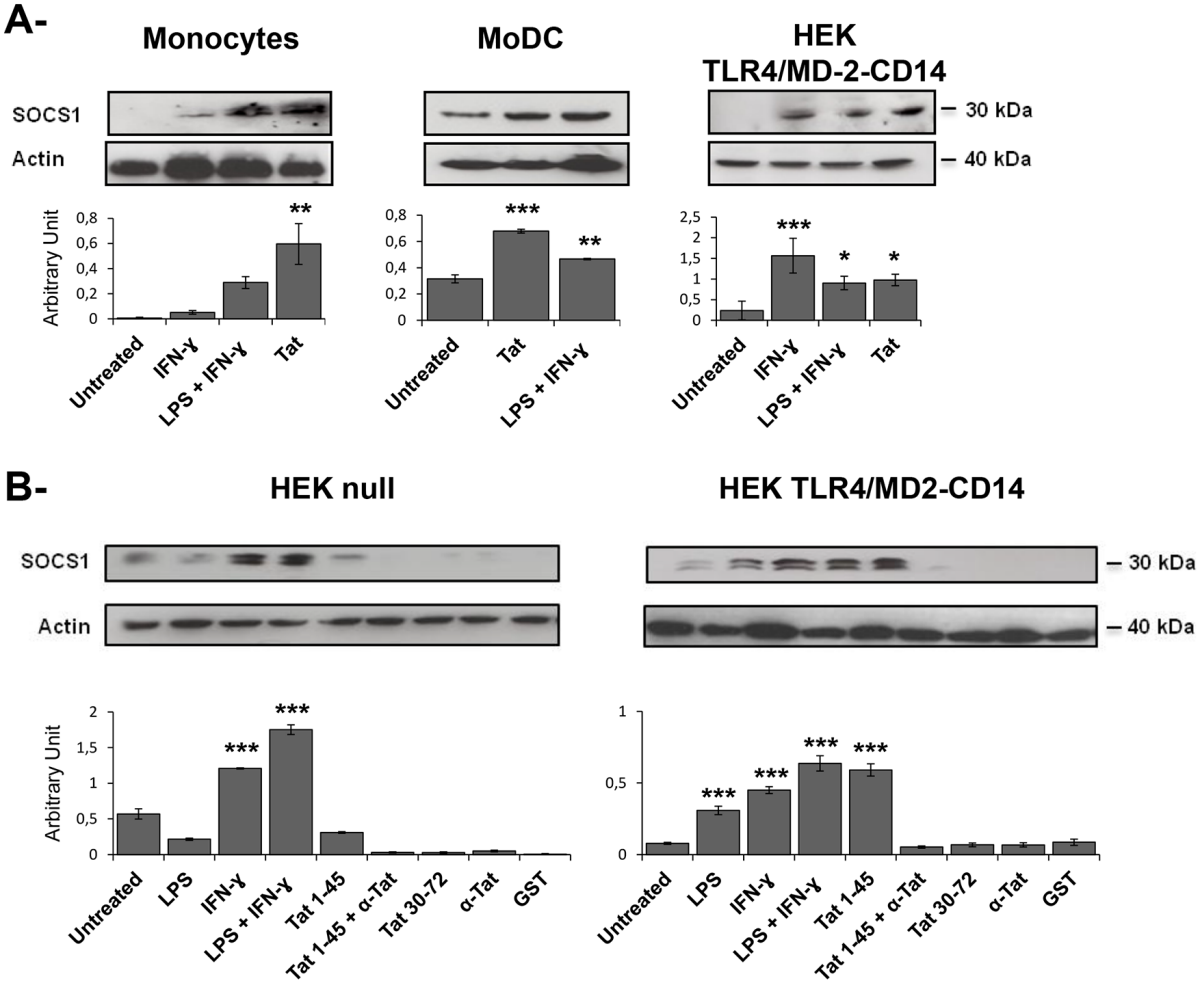
HIV-1 Tat induces SOCS1 expression. (**A**) Human monocytes, dendritic cells or HEK-TLR4-CD14-MD2 cells (10^6^) were stimulated or not for 24 h with Tat (100 nM) and tested for SOCS1 expression. LPS (10 ng/mL) or LPS + IFN-γ (10 ng/mL) were used as positive control. Actin was used as a loading control. (**B**) HEK-Null used as control or HEK cell line expressing TLR4-CD14-MD2 were stimulated with GST-Tat 1–45 (100 nM), GST-Tat 30–72 (100 nM) or LPS (10 ng/mL) +/- IFN-γ (10 ng/ml). The specificity of the Tat effect was tested in the presence of anti-Tat antibodies at 1 μg/ml. Stimulation with GST or with anti-Tat alone was used as controls. Quantification of the band obtained from 3 independent experiments was performed using Image J Software. Asterisks represent *P* values: *, *P ˂* 0.05; **, *P ˂* 0.01; ***, *P ˂* 0.001, ns non significant.

We next investigated whether the HIV-1 Tat protein recruited the suppressors of cytokine signaling (SOCS) pathway as an additional level of negative regulation of Tat-TLR4 activation. The family of SOCS proteins has 8 members (SOCS1 to SOCS7 and CIS). In addition to their involvement in the regulation of the TLR pathway [78], SOCS proteins are also involved in the attenuation of innate immunity. In particular, an increased level of SOCS1 protein has been associated with an increase of HIV-1 viral load and fast progression to AIDS [76,77,79,80]. Thus, we investigated whether the level of SOCS1 protein in human monocytes, DCs and HEK-TLR4-MD2-CD14 cell line was modulated following treatment with Tat protein. The level of induced SOCS1 protein was evaluated in cell extract after SDS-PAGE and western blotting using SOCS1 specific mAbs, as detailed in the *Materials and Methods*. As shown in Fig 7A and 7B, both Tat protein and its N-terminal fragment Tat 1–45, but not Tat 30–72 or GST alone, stimulated the expression of a significant amount of SOCS1 protein. SOCS1 protein was induced by Tat protein in TLR4-CD14-MD2 but not in HEK null cells (Fig 7B), thus further confirming the relationship between Tat-TLR4 interaction and SOCS1 induction. The induction SOCS1 protein was totally inhibited when the stimulation was performed in the presence of anti-Tat blocking mAbs (Fig 7B). As a positive control, we showed that SOCS1 was expressed in cells stimulated with LPS and/or IFN-γ (Fig 7A and 7B). Taken together, our results demonstrate that SOCS1 expression was induced following Tat-TLR4 interaction. Altogether the above results demonstrate that the HIV-1 Tat protein recruited the SOCS pathway as an additional level of negative regulation of Tat-TLR4 activation.

## Discussion

HIV-1 infection is associated with immunological dysfunctions characterized by an abnormal hyper-activation of the immune system, a selective depletion of CD4^+^ T-cells and an increase in the production of pro-inflammatory cytokines associated with fast progression of the infection towards AIDS [14–16,70,81,82]. The nature of HIV-1 proteins involved in these immunological dysfunctions and the underlying mechanism remain to be fully elucidated. We recently reported that the HIV-1 Tat protein induced expression of PD-L1 negative costimulatory molecule on DCs, through a TLR4 pathway, associated with a dysfunction in T-cell responses (42). However, whether and how HIV-1 Tat protein is involved in the abnormal hyper-activation and in the over-production of pro-inflammatory cytokines, such as IL-6 and IL-8, seen in HIV-1 infected patients, is unknown.

The present report is the first to demonstrate that HIV-1 Tat protein induces production of pro-inflammatory cytokines by DCs and monocytes/macrophages from HIV-1 infected subjects through engagement of the TLR4-MD2-CD14 complex, leading to overproduction of IL-6 and IL-8 pro-inflammatory cytokines by myeloid cells. To demonstrate that Tat induced IL-6 and IL-8 were produced by monocytes, but not by other cells from PBMC, we purified monocytes by positive selection and showed that only the CD14 positive fraction of PBMC was able to produce IL-6 and IL-8 following stimulation by recombinant or chemically synthesized Tat Protein. Moreover, IL-8 expression was detected at the intracellular level in CD14+ monocytes both *in-vitro* Tat-treated monocytes from healthy donor and Tat-Untreated monocytes from HIV-1 infected patients. Furthermore, the recruitment of TLR4-MD2-CD14 complex by Tat protein was demonstrated by the activation of TLR4 downstream pathways including NF-κB and SOCS-1 and by down-modulation of cell surface TLR4 by endocytosis in dynamin and lipid-raft-dependent manners. The study underlines the potential of HIV-1, *via* its early expressed Tat protein, to hijack the TLR4 pathway to establish an abnormal hyper-activation of the immune system. Such mechanism would contribute to immune dysregulation seen in HIV-1 infected patients and, hence, promote HIV-1 viral replication and progression to AIDS.

One of the potential viral factors involved in the abnormal immune responses seen in HIV-1 infected patients is Tat protein, in addition to gp120, Nef and Vpr HIV-1 proteins [18–20,22–31,83]. We and others have recently demonstrated that HIV-1 Tat protein is involved in the induction of various inflammatory cytokines including TNF-α [23,24,33,41,48,63,84,85], IL-β [25], IL-2 [58], IL-4 [24,86], IL-6 [62,87], IL-8 [40,88,89], IL-10 [22,33,45–47,60,61,64,90] and TGF-β [91–93] by acting on immune cells including B-cells, T-cells, monocytes, macrophages and DCs. Several groups have also shown that Tat protein stimulates various cytokines by non-immune cells including endothelial cells [89] and neurons [94]. Tat protein is present at nM levels in the serum of HIV-1 infected patients [55–57,95]. However, it is likely that the extracellular amount of Tat protein is underestimated, because the amounts detected in the serum did not take account of the proportion of Tat protein that might be adsorbed on the surface of cell membranes *via* heparan sulfates [96,97]. Moreover, the concentration of the extracellular Tat protein could be even much higher near the lymphoid organs and in the vicinity of infected cells [98]. Considering that Tat protein induced cytokine production by acting at the cell surface, several potential candidate receptors have been reported, including CD26 receptor [99], L-Type calcium channel [84,100], αvβ3 and α5β1 integrins [101,102], membrane lipids [103], the Flk-1/KDR receptor [104] and TLR4, as recently reported our group [33,42]. In this last work, we showed that Tat-induced TNF-α and IL-10 production in human monocytes is inhibited in the presence of blocking anti-TLR4 mAbs [33] and that Tat protein interacted in a solid phase assay with soluble recombinant TLR4-MD2 complex. However, it remained to directly demonstrate whether Tat-TLR4 interaction and its subsequent function effects on the activation of signaling pathways and cytokine production. In this study, we have shown that Tat protein is able to induce, specifically and in a dose-dependent manner, the production of IL-6 and IL-8 pro-inflammatory cytokines in both primary human monocytes and primary myeloid DCs. This result is in agreement with data previously reported by other groups [40,87–89], and suggests that HIV-1 Tat might be directly involved in over-stimulation of immune system and production of high level of inflammatory responses seen in HIV infected patients.

Our findings clearly show that both monocytes and MoDC isolated from HIV-1 infected patients produce a significant amount of IL-6 and IL-8 following Tat-stimulation. Interestingly, HIV-1 Tat protein also stimulated production of pro-inflammatory cytokines by monocytes and MoDC derived from healthy patients. As expected, individual variations, which are probably related to genetic polymorphisms, have been observed in both healthy subjects and HIV-1 infected patients. However, monocytes and MoDC obtained from HIV-1 infected patients produced lesser IL-6 and IL-8 than the same cells obtained from uninfected patients. These observations are in agreement with several others reports showing a significant decrease in the capacity of MoDC from HIV-1 infected patients to produce IL-12 and IL-10 [105] Similar observations reporting decrease responses of MoDC derived for HIV infected patients have been also reported [106] [107].

These IL-6 and IL-8 Tat-induced cytokines cannot be attributed to an indirect effect triggered by persistent viral replication, because no significant level of cytokines was release in the supernatant by non-Tat-treated monocytes and MoDC from HIV-1 infected patients. This observation is supported by the fact that all HIV-1 patients tested in this study (n = 11) were under active retroviral therapy and 7 of the 11 had undetectable viral load in the plasma with less than 20 copies of HIV-1 genome per mL of plasma. Moreover, in support of the latter observation, no significant levels of IL-6 or IL-8 cytokines were detected in the plasma of the eleven HIV-1 infected patients tested in this study (*data not shown*). However, intracellular staining for IL-8 and IL-6 in PBMC from HIV-1 infected patients with a detectable viral load reveal that a small population of monocyte produce IL-8 suggesting that *in-vivo* a viral factor induce IL-8 expression in monocytes. This result is in line with previous reports showing a direct relation between viral load and the pro-inflammatory cytokines present in the sera of HIV-1 infected patients [17,108]. Moreover, a progressive decrease of the levels of pro-inflammatory cytokines was noted in the sera of HIV-1 infected patients at the beginning of the anti-retroviral treatment (not shown), which could coincide with a diminution of Tat protein expression.

The over-production of IL-6 and IL-8, in combination with other pro-inflammatory cytokines, may contribute to an increase in HIV-1 viral replication and to a fast progression to AIDS. The IL-6 and IL-8, secreted by both HIV-1 infected and neighboring non-infected cells following stimulation by released extracellular Tat protein, may promote HIV-1 replication and progression to AIDS through several immune mechanisms. These include (*i*) modulating immune response, inflammation and hematopoiesis of B- and T-cell responses; (*ii*) increasing leukocyte recruitment; (*iii)* activating polyclonal B-cells; and (*iv*) causing lymphadenopathy, Kaposi sarcoma and lymphoma of the B cells [109–112]. While IL-6 participates in the activation of the HIV-1 promoter *via* NF-κB activation, IL-8, *via* its chemokine activity, may further participate by recruiting CD4^+^ cells, the targets of HIV-1, at the lymph nodes, which constitute a continuous site of viral replication [113,114]. Overall, our study, together with the previous reports mentioned above [33], highlight the importance of Tat-induced IL-6 and IL-8 pro-inflammatory cytokines in the physio-pathology of HIV-1 infection and AIDS.

Our study also presents strong results in line with our recent findings [33], demonstrating the engagement of TLR4-MD2-CD14 complex and activation of NF-κB Pathway by HIV-1 Tat protein, leading to IL-6 and IL-8 cytokine production. Complementary, direct approaches using anti-TLR4 blocking mAbs, HEK cell line stably transfected with TLR4-MD2-CD14, dominant negative and sh-RNA vectors targeting TLR4 and peritoneal macrophages isolated from mice KO for TLR4, strongly support the implication of Tat protein in the recruitment of the TLR4 pathway to induce the production of human IL-6 and IL-8 or murine IL-6 and CXCL1/KC cytokines/chemokines. Because the promoter organization of IL-6 and IL-8 contains NF-κB sites, we tested the capacity of Tat to activate NF-κB in primary human monocytes and HEK expressing TLR4-CD14-MD2 complex. First, we showed that this activation was strongly inhibited when the stimulation of primary human cells was performed in the presence of anti-TLR4 blocking mAbs. We subsequently showed that activation of NF-κB pathway by Tat protein occurred only in HEK cells expressing TLR4-CD14-MD2 complex, but not in HEK cells carrying an empty plasmid nor in HEK cells expressing TLR4 only, suggesting the strict requirement of TLR4-CD14-MD2 complex integrity for Tat to induce the production of pro-inflammatory cytokines. Moreover, HIV-1 Tat activated NF-κB pathway in peritoneal macrophages isolated from Wt. mice, but not from TLR4 KO mice, confirming the importance of TLR4 pathway.

In line with our findings, Uleri et al have recently reported that HIV-1 Tat protein is able to activate endogenous retroviruses of W family by acting on TLR4 receptor [115]. More interestingly the authors hypothesized a potential link between this effect and the neuropathogenic disorders observed in HIV-1 infected patients. Altogether, these results demonstrate that HIV-1 Tat engages the TLR4-MD2-CD14 complex and activates the NF-κB pathway leading to stimulation of IL-6 and IL-8 proinflammatory cytokines. Such a mechanism might contribute to the dysregulation of the immune response observed early in HIV-1 infection by hijacking TLR4 pathway to establish a proinflammmatory state which is inefficient for HIV-1 clearance and leads to AIDS pathogenesis development.

## Supporting Information

S1 Fig Tat induces IL-6 and IL-8 production in monocytes. (A) Monocytes were isolated from PBMC using either adherence protocol as described in Material and Methods, or positive selection using CD14 MicroBead according to the manufacturer's instructions (Miltenyi Biotec), CD14 negative fraction of PBMC was used as control. Cells were either kept untreated or treated with GST-Tat 1–101 (100nM). After 24h of incubation, cell supernatant was collected and use for cytokine quantification by ELISA as describes in Material and Methods. Results are expressed as means +/- SD. Differences in the means for the different groups was tested with Student's t test. Statistical significance are denoted with * for p < 0.05, ** p < 0.01, *** p < 0.001, ns not significant. (B) Monocytes fraction of PBMC was isolated by positive selection using CD14 MicroBead according to the manufacturer instruction (Miltenyi Biotec). Cells were either kept untreated or treated with GST-Tat 1–101 (10nM or 100nM) for 24hrs. Monensine 1X (GolgiStop) from BD Bioscience was added the last 6 hr. Cells were collected and stained with surface anti-CD14-APC (Biolegend) and intracellular anti-IL-8-PE (R&D) or Isotype control. Data were acquired on a FACSCalibur (BD). Results show CD14 surface expression *versus* intracellular IL-8 staining (left plots), and IL-8 staining in CD14+ monocytes (right side histogram).
(TIF)

S2 Fig Monocytes from HIV infected patients produce IL-8. PBMC were isolated from 6 different HIV infected donors with detectable viral load as described in Materials and Methods and incubated during 3h at 37°C in the presence of Monensine 1X (GolgiStop) from BD Bioscience. Cells were then collected and stained with surface anti-human CD3 (Pacific Blue) and anti-CD14 (APC) and intracellular IL-6 (FITC) and IL-8 (PE). Data were acquired on a Fortessa (BD). Plots are gated on CD14 and CD3 positive fraction of PBMC and the results show CD14 surface expression versus intracellular IL-8 (top line) or intracellular IL-6 (bottom line). CD14 negative cells correspond to CD3+ fraction of PBMC. (A) Shows the flow cytometry plots and (B) shows the percentage of monocytes (CD14+ fraction of PBMC) and T cells (CD3+ fraction of PBMC) producing IL-6 or IL-8, data are expressed as means +/- SD. Differences in the means for the different groups were tested with Student's t test. Statistical significance are denoted with *** for p < 0.001, ns not significant. (C) Shows one representative plot out of 3 independent experiments of the intracellular staining for IL-6 and IL-8 in PBMC from healthy donors.
(TIF)
