# Supplementary figures and images for: HIV-1 Tat Protein Induces Production of Proinflammatory Cytokines by Human Dendritic Cells and Monocytes/Macrophages through Engagement of TLR4-MD2-CD14 Complex and Activation of NF-κB Pathway
Source: PLoS One. 2015 Jun 19;10(6):e0129425. doi: 10.1371/journal.pone.0129425 (PMC4474861; doi:10.1371/journal.pone.0129425)

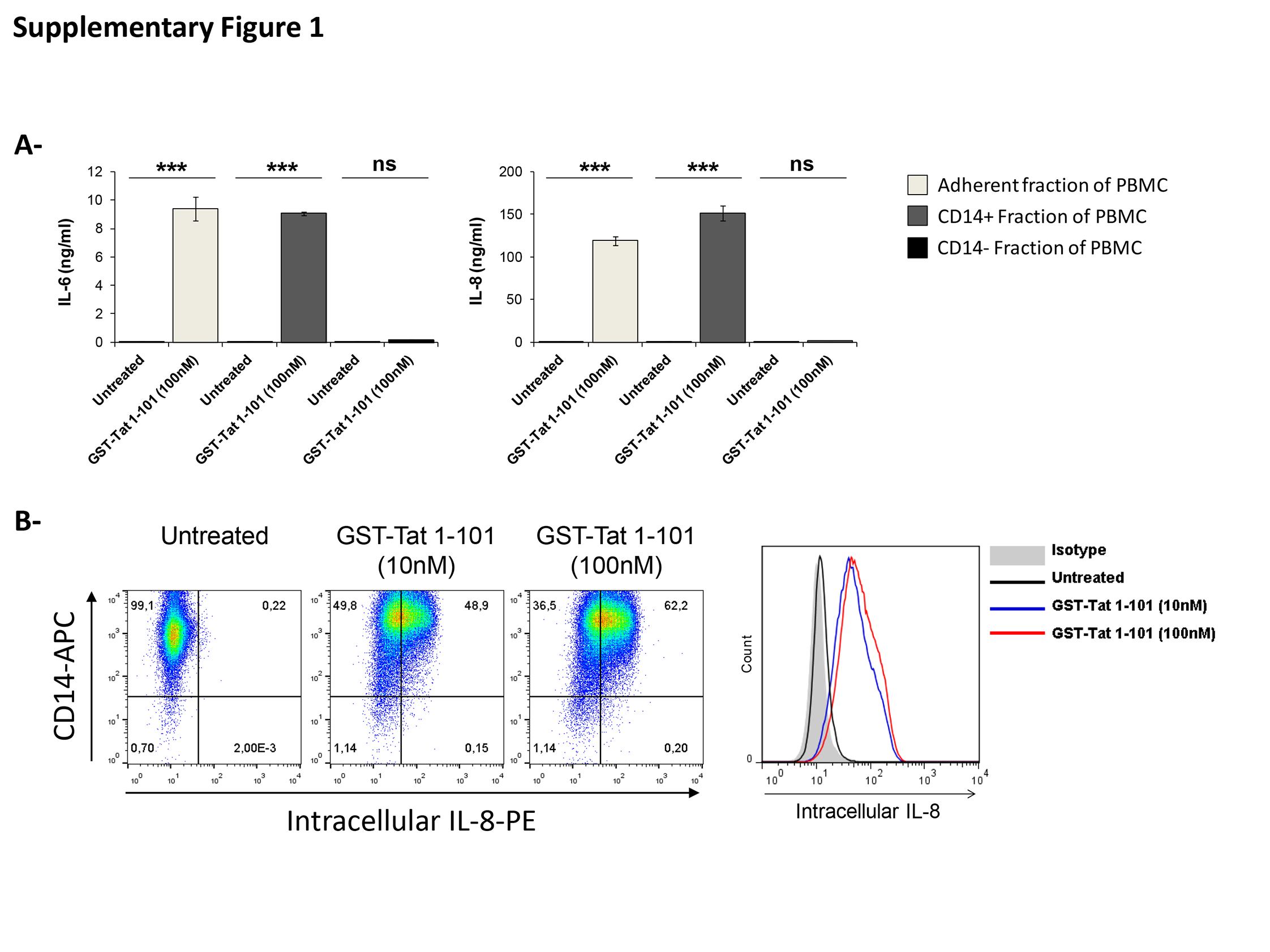

Supplement: S1 Fig — (A) Monocytes were isolated from PBMC using either adherence protocol as described in Material and Methods, or positive selection using CD14 MicroBead according to the manufacturer's instructions (Miltenyi Biotec), CD14 negative fraction of PBMC was used as control. Cells were either kept untreated or treated with GST-Tat 1–101 (100nM). After 24h of incubation, cell supernatant was collected and use for cytokine quantification by ELISA as describes in Material and Methods. Results are expressed as means +/- SD. Differences in the means for the different groups was tested with Student's t test. Statistical significance are denoted with * for p < 0.05, ** p < 0.01, *** p < 0.001, ns not significant. (B) Monocytes fraction of PBMC was isolated by positive selection using CD14 MicroBead according to the manufacturer instruction (Miltenyi Biotec). Cells were either kept untreated or treated with GST-Tat 1–101 (10nM or 100nM) for 24hrs. Monensine 1X (GolgiStop) from BD Bioscience was added the last 6 hr. Cells were collected and stained with surface anti-CD14-APC (Biolegend) and intracellular anti-IL-8-PE (R&D) or Isotype control. Data were acquired on a FACSCalibur (BD). Results show CD14 surface expression versus intracellular IL-8 staining (left plots), and IL-8 staining in CD14+ monocytes (right side histogram). (TIF) [file pone.0129425.s001.tif]

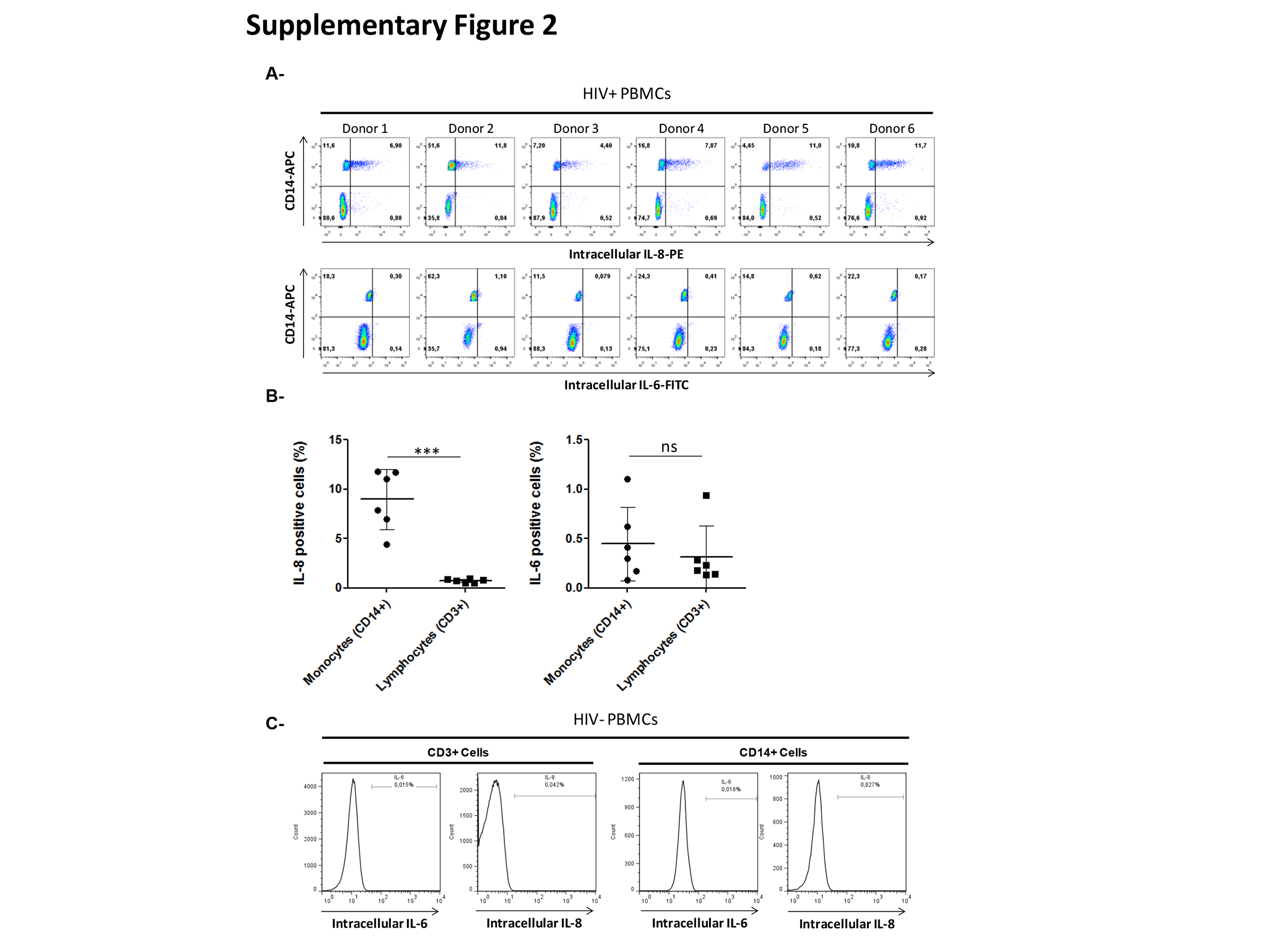

Supplement: S2 Fig — PBMC were isolated from 6 different HIV infected donors with detectable viral load as described in Materials and Methods and incubated during 3h at 37°C in the presence of Monensine 1X (GolgiStop) from BD Bioscience. Cells were then collected and stained with surface anti-human CD3 (Pacific Blue) and anti-CD14 (APC) and intracellular IL-6 (FITC) and IL-8 (PE). Data were acquired on a Fortessa (BD). Plots are gated on CD14 and CD3 positive fraction of PBMC and the results show CD14 surface expression versus intracellular IL-8 (top line) or intracellular IL-6 (bottom line). CD14 negative cells correspond to CD3+ fraction of PBMC. (A) Shows the flow cytometry plots and (B) shows the percentage of monocytes (CD14+ fraction of PBMC) and T cells (CD3+ fraction of PBMC) producing IL-6 or IL-8, data are expressed as means +/- SD. Differences in the means for the different groups were tested with Student's t test. Statistical significance are denoted with *** for p < 0.001, ns not significant. (C) Shows one representative plot out of 3 independent experiments of the intracellular staining for IL-6 and IL-8 in PBMC from healthy donors. (TIF) [file pone.0129425.s002.tif]
